# Transcriptome analysis of granulation tissue from periodontal osseous defects

**DOI:** 10.1002/JPER.24-0821

**Published:** 2025-05-12

**Authors:** Ye Han Sam, Ana J. Caetano, Dewi R. Owen, Giuseppe Mainas, Luigi Nibali, Mandeep Ghuman

**Affiliations:** ^1^ Periodontology Unit, Centre for Host‐Microbiome Interactions Faculty of Dentistry, Oral and Craniofacial Sciences King's College London London UK; ^2^ Centre of Periodontology Studies, Faculty of Dentistry MARA University of Technology, Universiti Teknologi MARA Sungai Buloh Sungai Buloh Selangor Malaysia; ^3^ Institute of Dentistry Faculty of Medicine and Dentistry Queen Mary University of London London UK

**Keywords:** granulation tissue, periodontitis, regeneration, RNA sequencing, wound healing

## Abstract

**Background:**

Granulation tissue is routinely discarded in periodontal surgical procedures, but few studies have characterized it. The present study aimed to compare global gene expression in granulation tissue derived from different types of periodontal osseous defects.

**Methods:**

Total RNA was isolated from granulation tissue harvested during routine periodontal surgical procedures. Nineteen sites were analyzed—seven infrabony, six suprabony, and six furcation defects. Following quality control checks, samples underwent bulk mRNA sequencing (20–30 million read pairs per sample) before bioinformatic analyses utilizing gene ontology and associated pathway/enrichment analyses.

**Results:**

No statistically significant differentially expressed genes (DEG) were detected between different osseous defect types. An increase in the expression of 11 genes with a false discovery rate of <0.05 was detected when a comparison was made in terms of healing duration post nonsurgical periodontal therapy (NSPT). Notably, the genes involved included those regulating collagen synthesis and osteogenic activity. Analysis based on sex differences revealed 38 DEG. Gene enrichment analysis revealed that 24 DEG without Y‐linked attachment are mostly involved in immune regulation.

**Conclusions:**

Routinely discarded periodontal granulation tissue exhibits epithelial characteristics due to a substantial period of maturation post NSPT. Confirmation of ongoing maturation beyond traditional periodontal re‐evaluation timepoints warrants further investigation on tissue differentiation potential. More research is needed to elucidate the role of sex as a biological variable affecting periodontal immune response.

**Plain language summary:**

Granulation (wound) tissue is routinely removed during gum (periodontal) surgical procedures, but knowledge on its characteristics is scarce. This study aimed to compare gene expression in granulation tissue derived from different types of periodontal bone defects via high‐throughput RNA sequencing. Total RNA was extracted from 95 samples harvested from gum disease patients during surgery. After quality control checks, 19 samples (seven infrabony, six suprabony, and six furcation defects) were eligible for sequencing. Subsequent analyses were done utilizing software with known cell biological pathways and processes. Analysis revealed no differentially expressed genes (DEG) in terms of periodontal defect category. There was statistically significantly increased expression of 11 genes when a comparison was made in terms of healing duration following deep scaling treatment. These genes are involved in collagen synthesis and osteogenic activity. Interestingly, analysis based on sex differences detected 38 DEG. Gene enrichment analysis revealed that 24 DEG without association with Y chromosomes are mostly involved in the regulation of immune system response. Routinely discarded periodontal granulation tissue exhibits lining cell characteristics that change over time following deep scaling treatment. More research is needed to unravel the role of sex as a biological variable affecting periodontal immune response in this type of tissue.

## 1 INTRODUCTION

Based on cutaneous wound research models, periodontal wound healing consists of four overlapping phases: hemostasis, inflammation, granulation, and remodeling.^1^ Tissue reorganization commences during the granulation phase whereby immunomodulatory macrophages orchestrate new tissue construction within the wounded region.^2^ This results in the formation of granulation tissue, a highly vascularized tissue with fibroblasts as its principal cellular component accompanied by inflammatory cells embedded in a fibrin‐rich extracellular matrix (ECM).^1^ Disruption to ideal maturation of granulation tissue leads to undesirable wound repair resulting in suboptimally remodeled tissue with compromised mechanical properties.^3^

Emerging evidence has revealed differences in wound healing between human skin and oral mucosa,^4^ but the molecular basis of their distinctiveness remains to be fully elucidated, including the characterization of granulation tissue. Granulation tissue is routinely discarded during surgical management of periodontal defects with the intention of facilitating optimum wound healing and space provision for regeneration.^5^ Conversely, novel surgical approaches incorporating the preservation of granulation tissue have also been suggested based on harnessing its regenerative potential due to putative stem cell‐like properties.^6^, ^7^, ^8^ A recent study^9^ has also shed light on the levels of cell signaling molecules associated with proresolving macrophage phenotypes from within periodontitis‐affected granulation tissue, namely IL‐10, CD163, and tumor necrosis factor‐like weak inducer of apoptosis (TWEAK), which may influence wound healing post nonsurgical therapy.

The advent of next‐generation sequencing technologies has allowed for more sensitive characterization of human tissue under diseased and health conditions. Its application has enabled a greater understanding of transcriptomic differences that may reveal potential therapeutic targets. In this study, we aimed primarily to compare the transcriptional differences between granulation tissue from different periodontal osseous defects. Considering the influence of time in wound healing response, a comparison was also made in terms of different reassessment timepoints following nonsurgical periodontal therapy (NSPT). Furthermore, the uniqueness in immune regulation between males and females provided the basis for the investigation of the transcriptional profile in terms of sex differences.

## 2 MATERIALS AND METHODS

### 2.1 Patient selection

The study falls under the Dental, Oral, and Craniofacial Biobank Study (Integrated Research Application System [IRAS] no. 275079). National Health Service (NHS) ethics approval was obtained from the East of England – Cambridge East Research Ethics Committee (reference 20/EE/0241) and approval from the Biobank Management Committee of Guy's Hospital London (access form 006, approved on December 1, 2022). Patients were invited to participate from within the Periodontology Unit of Guy's Hospital, London. Included in the study were periodontitis patients (diagnosed according to the latest classification)^10^ who (1) had given written and verbal consent to the above study, (2) were systemically healthy, (3) were not taking any medication that could influence periodontal treatment, and (4) had an indication to undergo a periodontal surgical procedure based upon the standard of care complying with the S3 clinical treatment guidelines by the European Federation of Periodontology.^11^ Patients who were (1) smokers and (2) had poor plaque control needing further reinforcement of Step 1 and Step 2 of periodontal therapy were excluded. All samples were harvested from patients undergoing surgical treatment from January 2023 to January 2024.

This observational study complied with the guidelines for Strengthening the Reporting of Observational Studies in Epidemiology (STROBE).^12^ Due to the exploratory nature of the study, a convenience sampling approach was taken in accordance with the inclusion and exclusion criteria stipulated in the Biobank protocol.

### 2.2 Tissue sampling

Granulation tissue samples were harvested from patients who had undergone at least one course of NSPT. NSPT was carried out carefully with a combination of ultrasonic scalers and periodontal curettes with no intentional removal of granulation tissue. Clinical operators were postgraduate residents specializing in periodontology, and all surgical procedures were carried out under the supervision of qualified specialists.

The minimum volume of all samples exceeded 8 mm^3^ and was categorized based on periodontal osseous defect morphology. The location of the base of the periodontal pocket in relation to the alveolar crest forms the basis of defect classification as follows:

1. Suprabony defect: An osseous defect where the base of the pocket is above the crestal height.
2. Infrabony defect: An osseous defect where the base of the pocket is apical with respect to the crestal height (≥3 mm depth of infrabony component).
3. Furcation defect: An osseous defect that is localized in the anatomical region of a multirooted tooth where root divergence occurs (horizontal furcation degree ≥2).

As the samples were harvested at different timepoints following NSPT, they were further categorized in terms of healing period post NSPT as follows:

1. Samples collected <6 months following NSPT
2. Samples collected ≥6 months following NSPT

To ensure that only granulation tissue was harvested, several measures were taken. First, care was taken to avoid harvesting the adjacent epithelial component, more specifically the pocket epithelium, so that the deeper tissue was prioritized to avoid the inclusion of nonaffected gingival tissue. Second, through washing the samples with sterile phosphate buffered saline (PBS), gingival epithelium that might have been erroneously included during the harvesting process was discriminated and removed on the basis of the vascular nature of the granulation tissue. Additional parts of granulation tissue were discarded if the presence of lining epithelium was observed or suspected. For samples harvested from infrabony defects, care was ensured to avoid inclusion of the suprabony component using the bone crest as a reference point.

Upon removal, the harvested samples were stored immediately in cold PBS solution within sterile Eppendorf tubes. After washing, the samples were fixed within an RNA stabilization solution* and stored frozen under −80°C for later RNA extraction.

### 2.3 Total RNA isolation and purification

Frozen samples were first thawed before being minced into smaller sizes using disposable sterile scalpel blades on sterile petri dishes. RNA was extracted using an RNA extraction kit† according to the manufacturer's protocol. Subsequently, the extracted RNA concentration and purity (260/280‐ and 260/230‐nm ratios) were measured using a spectrophotometer.‡ RNA integrity was assessed using an automated electrophoresis system§ according to the manufacturer's protocol.

### 2.4 Library preparation for RNA sequencing

RNA sequencing was applied, and library construction with 20–30 million reads per sample was done using a next‐generation sequencing system‖ at the Genome Centre of Queen Mary University of London. Raw reads were aligned to the human reference genome GRCh38/hg38. Raw sequence data (.bcl files) were demultiplexed and converted to text‐based file format (.fastq files) for downstream bioinformatic analysis.

### 2.5 Bioinformatic analysis

The quality control of raw reads was assessed using FastQC (v0.12.1). Adapter sequences were removed using Cutadapt (v4.9). Reads were aligned to the human reference genome GRCh38/hg38 using RNA STAR (v2.7.11a). The quantification of reads per gene was derived using FeatureCounts (v2.0.3). The DESeq2 (v2.11.40.8) package was used to conduct differential expression analysis of the RNA sequencing data. Normalized gene counts with their respective *p* values, false discovery rates (FDR), and fold change were then tabulated. Genes that satisfied a log2fold change >0.5 and FDR <0.05 were considered differentially expressed. Genes of interest were uploaded onto the Database for Annotation, Visualization, and Integrated Discovery (DAVID) for Gene Ontology (GO), and Kyoto Encyclopedia of Genes and Genomes (KEGG) and Reactome pathway analyses carried out to understand the relevant biological processes and pathways.

## 3 RESULTS

### 3.1 Sample characteristics

Total RNA was extracted from 95 harvested samples. After filtration of samples recording low concentrations with unsuitable purity ratio, 75 were selected for RNA integrity assessment (RIN). Based on the overall evaluation of RNA concentration, purity, and quality (RIN), a total of 19 samples from 18 patients were eligible for RNA sequencing and bioinformatic analysis. The analyzed granulation tissues were harvested from six suprabony defects, seven infrabony defects, and six furcation defects. Two different samples based on defect category harvested from two separate sites (one suprabony, one infrabony) were obtained from a single patient. The remaining samples were obtained from individual patients. Samples were almost proportionately distributed according to sex (10 men, 9 women) and harvesting time (<6 months post NSPT = 10; ≥6 months post NSPT = 9). No major intragroup differences were observed in terms of probing pocket depth (PPD) and clinical attachment loss (CAL) recorded at baseline and before surgery. Most samples were collected from multirooted teeth with PPD and CAL within the range of 6–8 mm, with a handful from sites registering ≥9 mm. Table 1 provides the summary of sample characteristics that were sent for sequencing and analysis.

**TABLE 1:** Sample characteristics at time of harvesting.

Variable	Distribution (*n*)
Total number of patients	18
Total number of samples	19
Periodontitis diagnosis	
Stage	
Stage III	9
Stage IV	10
Grade	
Grade C	19
Extent	
Generalized	18
Localized	1
Age	
<30	1
30– < 40	5
40– < 50	4
>50	8
Sex	
Male	10
Female	9
Defect morphology	
Suprabony	6
Infrabony	7
Furcation	6
Time of harvesting	
<6 months following NSPT	10
≥6 months following NSPT	9
PPD at baseline (before NSPT)	
6–8 mm	12
≥9 mm	7
CAL at baseline (before NSPT)	
6 –8 mm	5
≥9 mm	14
PPD before surgery	
6–8 mm	14
≥9 mm	5
CAL before surgery	
6–8 mm	6
≥9 mm	13
BOP	
BOP at baseline	19
BOP before surgery	19
Tooth type	
Single‐rooted teeth	2
Multirooted teeth	17

*Note*: PPD and CAL were recorded based on deepest site.

Abbreviations: BOP, bleeding on probing; CAL, clinical attachment loss; NSPT, nonsurgical periodontal therapy; PPD, probing pocket depth.

### 3.2 Gene expression profiling by defect morphology

**FIGURE 1:**
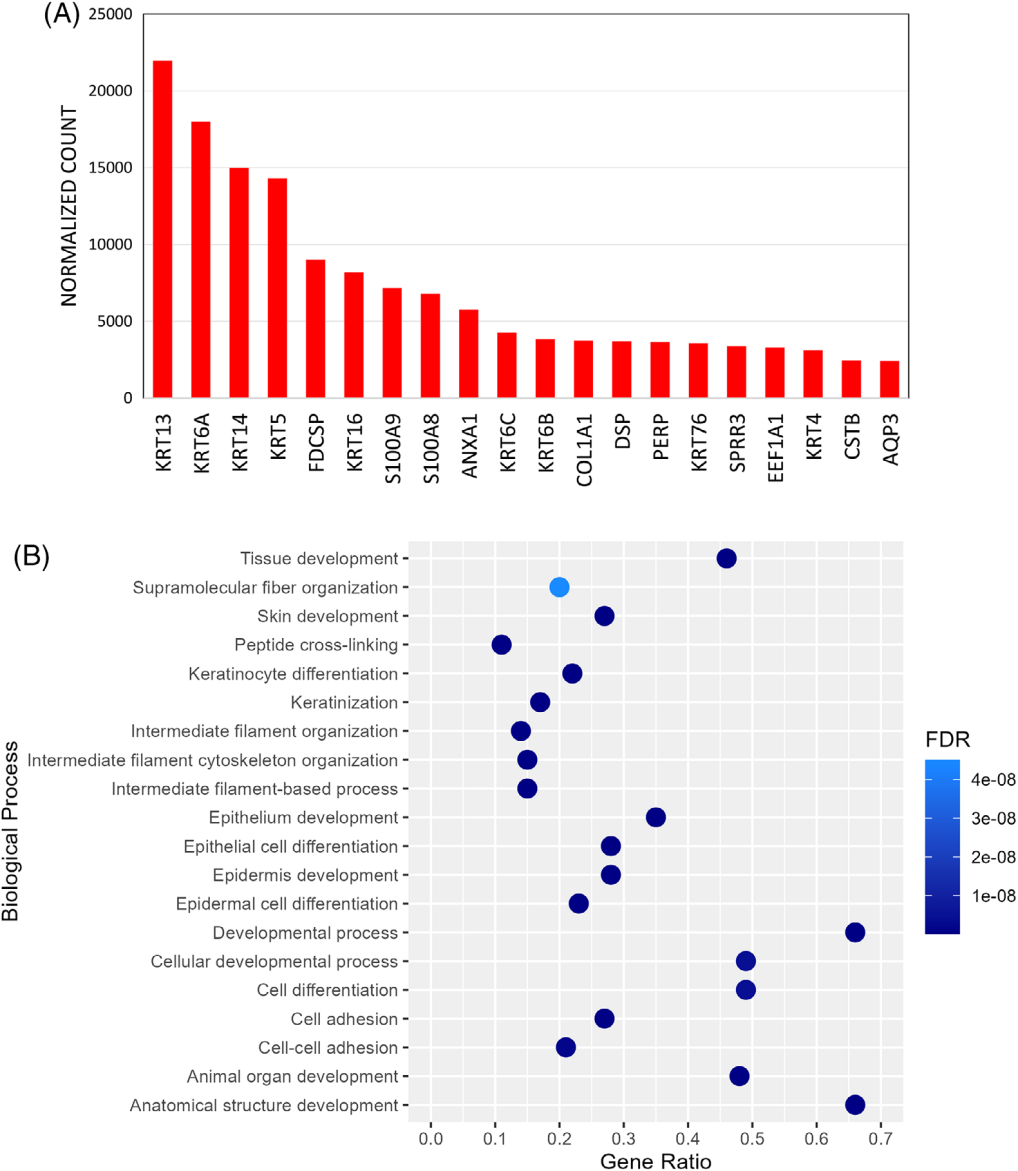
Gene profile of top 20 most highly expressed genes across all defect morphologies ranked by normalized gene count. (A) Bar chart of expressed genes indicated by normalized gene count averaged across all samples. (B) Scatter plot of GO‐annotated biological processes generated using gene ratio and FDR values specific to collective analysis of genes. FDR, false discovery rates; GO, gene ontology.

Across the three defect categories, a total of 39,077 genes were identified without genes unique to a defect category. Based on pairwise comparisons by defect morphology, no differentially expressed genes (DEG) were detected at a log2fold change >0.5 and FDR <0.05. Figure 1 shows the top 20 most highly expressed genes, ranked by normalized gene count, revealing that the most prominently expressed genes are involved in the synthesis of proteins relevant to structural components of epithelial cells and participate in immunoregulatory functions. GO annotation revealed biological processes related to the differentiation of epithelial components and maintenance of its structural integrity.

### 3.3 Gene expression profiling in terms of healing period following NPST

**FIGURE 2:**
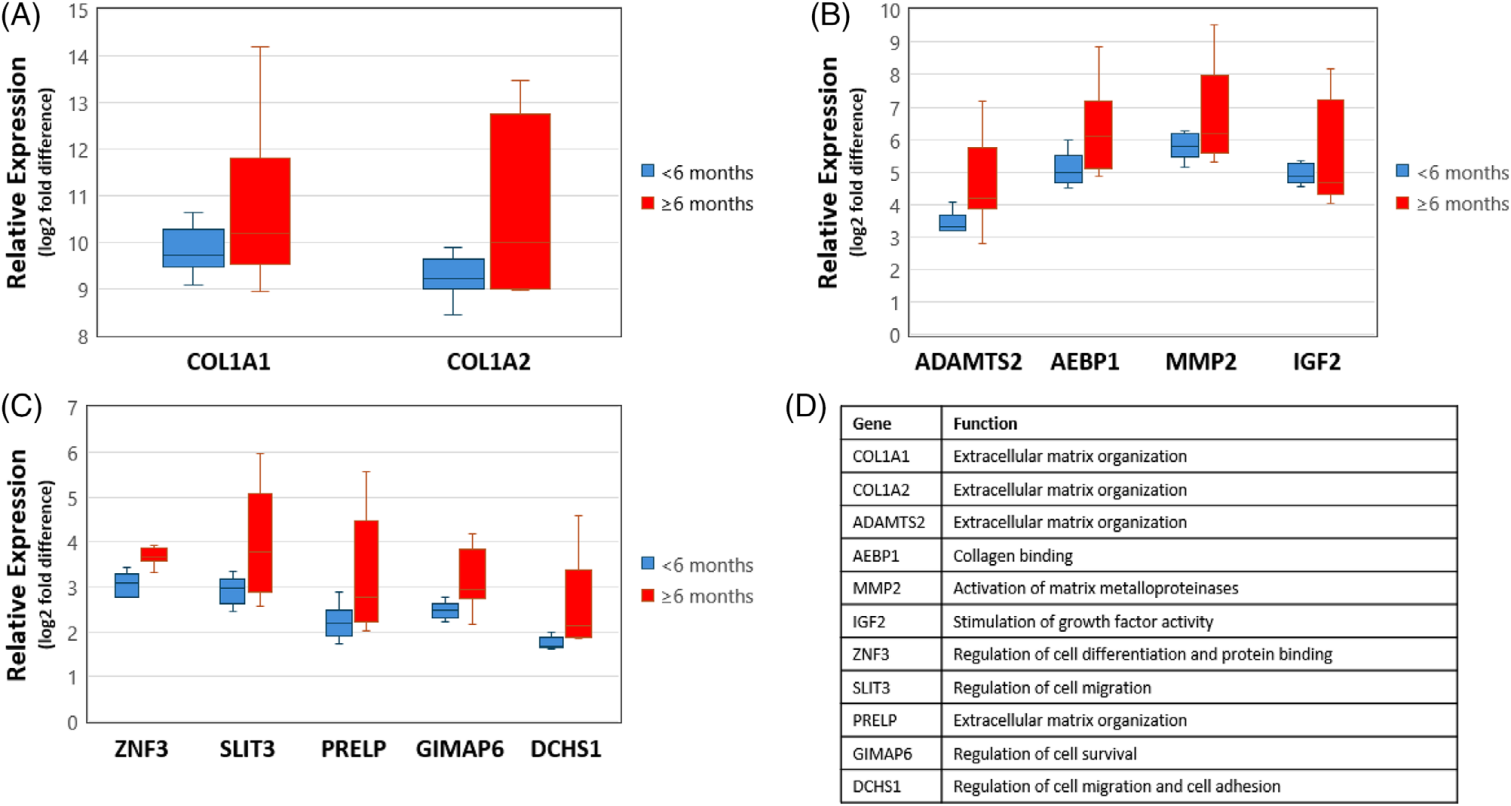
Analysis of expression of genes at FDR < 0.05 when compared over two timepoints of harvesting (<6 vs. ≥6 months after completion of NSPT). Box plots in Figures 2A–C were generated by comparing relative expression of genes with log2 fold difference between two timepoints. Figure 2D lists genes with corresponding functions. FDR, false discovery rates; NSPT, nonsurgical periodontal therapy.

**FIGURE 3:**
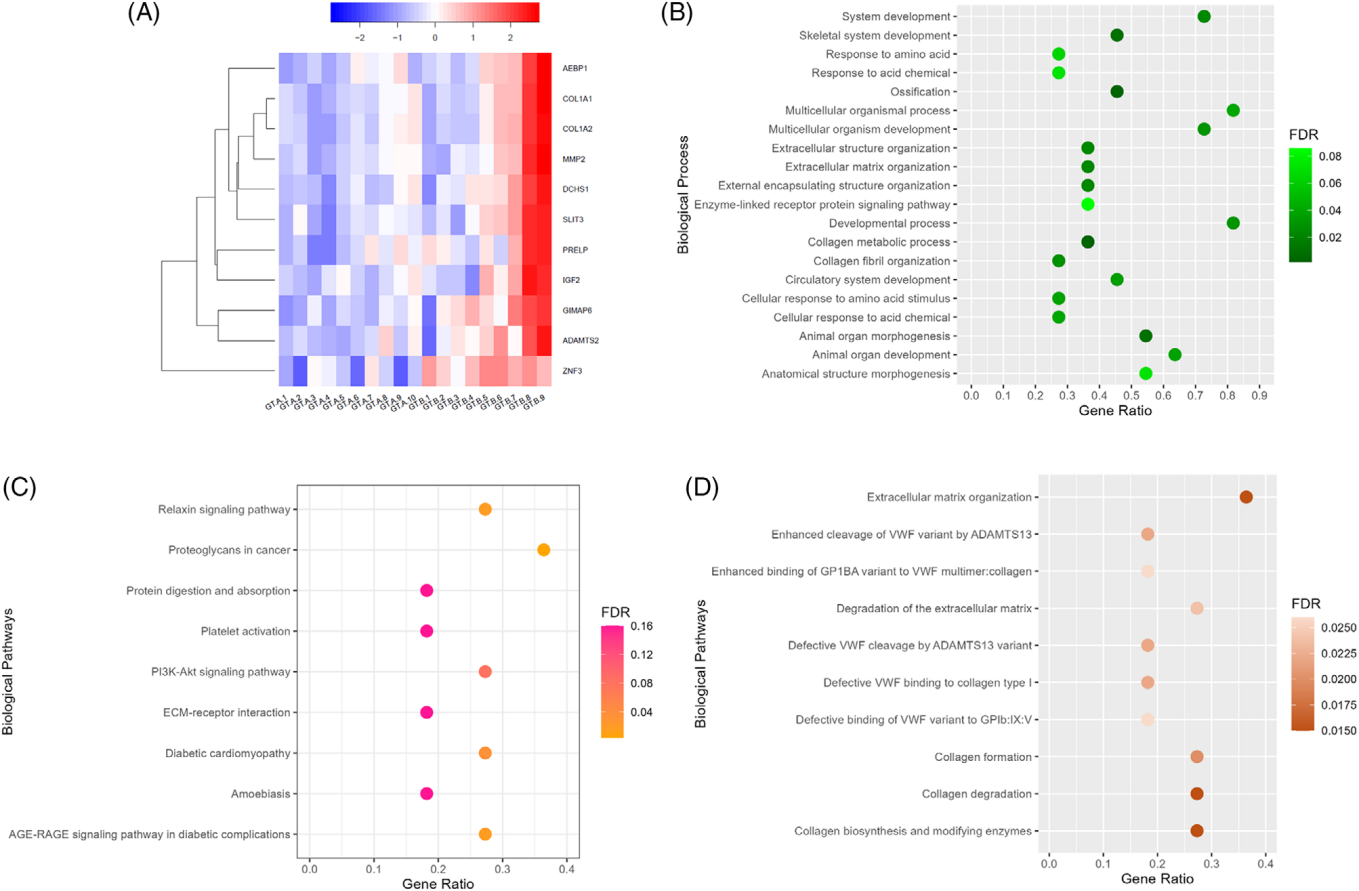
Collective analysis of genes expressed at FDR < 0.05 when compared over two timepoints of harvesting (<6 months vs. ≥6 months post NSPT). (A) Heatmap of annotated genes between samples belonging to two timepoints. (B) Scatter plot of GO‐annotated biological processes upregulated at later post‐NSPT healing period. (C) Scatter plot of KEGG‐annotated pathways upregulated at later post‐NSPT healing period. (D) Scatter plot of Reactome‐annotated pathways upregulated at later post‐NSPT healing period. Gene abundance was generated using DESeq2 (v2.11.40.8) and annotated via GRCh38 reference genome. Scatter plots were generated using gene ratio and FDR values specific only to collective analysis of genes. FDR, false discovery rates; GO, gene ontology; GT‐A, granulation tissue harvested at <6 months following completion of NSPT; GT‐B, granulation tissue harvested at ≥6 months following completion of NSPT; KEGG, Kyoto Encyclopedia of Genes and Genomes; NSPT, nonsurgical periodontal therapy.

Next, gene expression profiles were compared between two timepoints of the healing period using a 6‐month demarcation following completion of NSPT prior to surgical intervention: <6 months post NSPT healing (*n* = 10, average of 18.7 weeks) and ≥6 months post NSPT healing (*n* = 9, average of 34.8 weeks). Pairwise comparison of samples from the two timepoints identified 11 genes exhibiting FDR < 0.05, including *COL1A1, COL1A2, ADAMTS2, AEBP1, MMP2* *, IGF2, ZNF3, SLIT3, PRELP, GIMAP6*, and *DCHS1*. Figure 2A–D shows that the 11 genes increased in expression from samples collected at a later healing period. *COL1A1* and *COL1A2* were the two most highly expressed genes. The heatmap in Figure 3A shows the comparison of the gene expression between the two timepoints and the corresponding GO‐annotated biological processes (Figure 3B). Besides biological activity related to the restoration of tissue architecture, processes relevant to bone mineralization were also expressed. Concurrently, KEGG analysis (Figure 3C) revealed pathways related to angiogenesis and vascular functions (platelet activation; relaxin signaling pathway), structural reorganization (ECM receptor interaction; protein digestion and absorption), regulation of cellular proliferation (PI3K‐Akt signaling pathway; proteoglycans in cancer), and inflammation‐inducing pathological conditions (AGE‐RAGE signaling pathway in diabetic complications; diabetic cardiomyopathy; amoebiasis). Additional Reactome pathway analysis (Figure 3D) provided further information on ongoing connective tissue differentiation, specifically on the activity of collagen proteins.

### 3.4 Gene expression profiling based on sex differences

**FIGURE 4:**
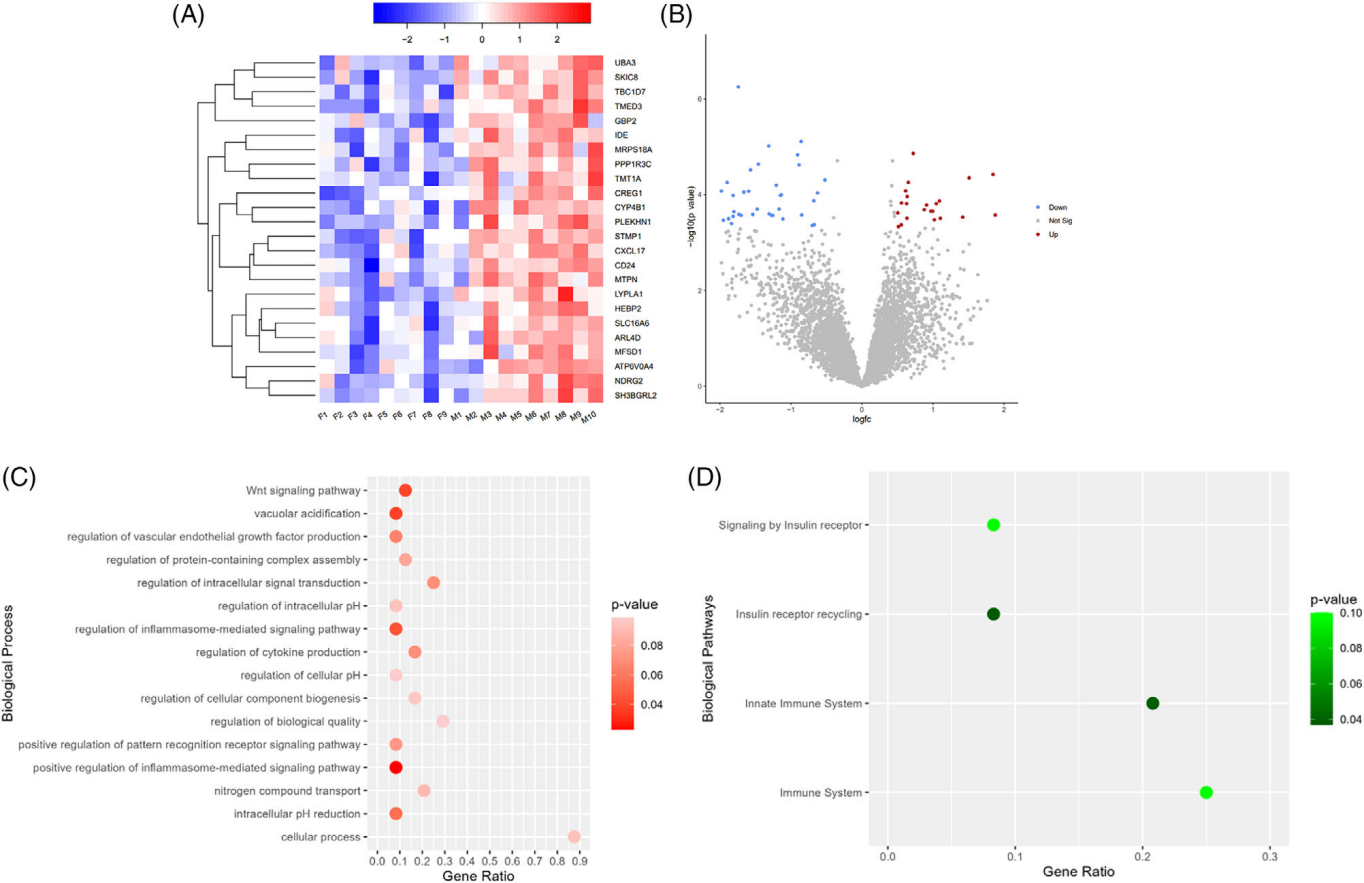
Collective analysis of DEG comparing female and male samples. (A) Heatmap of DEG (female vs. male). (B) Volcano plot of DEG (female vs. male) with red dots indicating upregulated genes in males and blue dots indicating downregulated genes. (C) Scatter plot of GO‐annotated biological processes upregulated in males. (D) Scatter plot of Reactome‐annotated pathways upregulated in males. Scatter plots were generated using gene ratio and *p* values specific to collective analysis of genes. Y‐linked genes were excluded from enrichment analyses. DEG, differentially expressed genes; F, female; GO, gene ontology; M, male.

An additional investigation was conducted using sex as a variable, which resulted in the detection of 38 DEG. Fourteen genes were identified as Y‐linked, which are unique to males. Figure 4 provides further details on the remaining 24 DEG, which were coexpressed in both sexes. The heatmap in Figure 4A and the volcano plot in Figure 4B provide a concise breakdown on the downregulation and upregulation of DEG based on sex differences. GO annotation (Figure 4C) revealed that the most dominant biological processes are largely related to immune response. No KEGG pathway analysis could be generated using this set of genes, while Reactome pathway analysis (Figure 4D) revealed pathways representative of immune signaling.

## 4 DISCUSSION

The present exploratory study analyzed what is classically and clinically referred to as periodontal granulation tissue^1^ via high throughput RNA sequencing. From a statistical standpoint,^13^ no DEG were detected when a comparison was made based on defect morphology as none could simultaneously satisfy the two suggested thresholds for fold change and FDR. Thus, the anatomical architecture of periodontal defects in the present study does not seem to influence the transcriptional profile of granulation tissue. Granulation tissue from periodontal defects can derive from different anatomical components, therefore it can be speculated that suprabony and infrabony components can have different gene expression profiles. However, through molecular profiling of gingival crevicular fluid, suprabony and infrabony defects do not appear to possess unique microenvironments representing distinct pathogenetic continuums.^14^ Interestingly, the same group reported distinctively higher levels of inflammatory and wound repair markers from furcation defects, which could be due to its more complex morphology.^15^ Although it can be speculated that a distinct wound healing profile may be present within furcation defects, molecular signatures specific to particular timepoints may be insufficient to unravel the complex wound healing process leading to the formation of granulation tissue, especially post NSPT. Transcriptomic profiling across all defects revealed that the dominantly expressed genes are involved in keratin synthesis and epithelial differentiation,^16^ namely, *KRT4*, *KRT5*, *KRT13*, and *KRT14*. This corresponds with the GO‐enriched biological processes suggesting that the samples exhibit epithelial characteristics. Following NSPT, there is evidence suggesting the completion of epithelial proliferation even within 7 days.^17^ Within the wound healing continuum, the granulation phase transitions to the remodeling phase no later than 4 days, lasting up to 3 weeks.^3^ From the existing patient pool, surgeries were performed in sites no less than 3 months after NSPT in compliance with the latest guidelines.^11^ This longer healing period may have affected the degree of maturation of the samples, which suggests that periodontal granulation tissue is a chronic wound tissue that has undergone a noticeable degree of remodeling. As sites with deep PPD and BOP indicate disease progression,^18^ the chronic inflammatory process within such tissues is unlikely to have ceased during time of harvesting because bleeding was detected on all sampled sites indicative of active inflammation. The prominent expression of *S100A8*, *S100A9*, *FDCSP*, and *ANXA1* may also suggest the tissue's involvement in immunomodulation^19^, ^20^, ^21^ via molecular actions of inflammatory cytokines, macrophages, and T and B lymphocytes.

Although pocket closure is achievable in moderately deep PPD,^22^ the present study reported a similar distribution of the baseline and presurgical PPD range. Despite undergoing NSPT, only a minimal impact on baseline PPD reduction was achieved in the harvesting sites, which were predominantly multirooted teeth. Surgery was in fact carried out in these nonresponding sites. Multirooted teeth have been reported to demonstrate a poorer response to NSPT due to the more complex anatomical profile.^23^ Conventionally, periodontal re‐evaluation at a 2‐ or 3‐month timepoint has been suggested to optimally assess healing response to NSPT.^24^, ^25^ Several studies have compared 3‐month and 6‐month timepoints in the investigation of pocket closure.^26^, ^27^ The effects of NSPT have also been documented even beyond 9 months.^28^ Considering the scarcity in reporting healing response beyond 6 months post NSPT, a 6‐month demarcation was selected to enable further understanding of tissue maturation with respect to time. The comparison paved the way for the detection of a set of genes with FDR < 0.05, asserting time as a critical variable affecting tissue maturation. Of relevant interest, *COL1A1* and *COL1A2*, which encode proteins for collagen synthesis, are the two most highly expressed genes in this context. The remodeling of wounded tissue can extend up to 12 months with continued deposition of collagen, specifically type I collagen.^29^ Fibroblasts are the principal cells of wound repopulation, and oral fibroblasts have been demonstrated to possess a better repopulation capacity than dermal fibroblasts.^30^ Therefore, oral wounds have been postulated to heal faster, with more favorable outcomes than dermal wounds.^31^ A rapid healing rate in oral wounds may suggest earlier resolution, but the present study may indicate ongoing deposition of structural components, including collagen, for a longer period.

The time‐related comparison also revealed a significant increase in the expression of *MMP2*. A clinical study^32^ has reported a nonsignificant reduction in *MMP2* levels despite receiving NSPT with adjunctive systemic antibiotics, which could be attributed to the enzyme's role in tissue remodeling during wound healing. This elevated expression may suggest that the resolution of periodontal deterioration has not been fully achieved considering *MMP2*′s role as a biomarker for periodontal destruction. This further re‐emphasizes the chronicity of periodontal granulation tissue due to impaired wound healing.

It has been documented that *MMP2* plays a pivotal role in processing type I collagen, which contributes to the coupling mechanism of bone formation and resorption in skeletal development.^33^ Deficiency in *MMP2* is associated with impaired organization of bone matrix, resulting in poorer biomechanical properties of bone.^30^ Nevertheless, the collagen‐cleaving properties of *MMP2* may interrupt ideal collagen deposition if the enzyme is overexpressed within the wounded tissue.^34^ The simultaneous elevation of *COL1A1*, COL1A2, and *MMP2* may reflect an elevated level of connective tissue deposition.

Furthermore, the detection of *SLIT3*, *PRELP*, and *IGF2* at FDR < 0.05 is also an interesting observation as expression of these genes may broadly imply pro‐osteogenic activity. *SLIT3* has been reported to be highly expressed in osteoblasts, and its proangiogenic characteristics augment the formation of skeletal vascular endothelium, which helps in regulating bone formation.^35^ *PRELP* provides instructions for synthesis of a leucine‐rich repeat protein, which has been reported to interfere with NF‐κB signaling resulting in impaired osteoclastogenesis.^36^ An in vivo investigation has also reported *PRELP* as a potential regulator of osteoblastic differentiation through its involvement with the β‐catenin signaling pathway.^37^ *IGF2* has been suggested to exert a synergistic effect on BMP‐9 resulting in bone matrix mineralization.^38^ Interestingly, *IGF2* and BMP‐9 may cross‐regulate through the PI3K/AKT signaling pathway, which has been annotated in the above KEGG pathway analysis. Therefore, it can be hypothesized that osteogenic activity increases significantly in a later healing period post NSPT. The existing data correspond well with two recent studies which reported on the remodeling capacity of inflamed soft tissue around residual periodontal pockets. The expression of genes promoting tissue reorganization and collagen deposition suggests the ongoing role of granulation tissue in wound healing with the potential for further proliferation to reconstruct damaged sites.^39^, ^40^ This potential for further healing contrasts a clinical study that reported a reduction in immunocompetent cells within the regenerated gingival tissue on sites receiving surgical debridement without soft tissue resection.^41^ Nevertheless, a recent meta‐analysis has surmised that a resective and open‐flap approach with no soft tissue resection does not result in clinical differences in terms of PPD reduction after 3 years.^42^ One of the samples was harvested from a relatively young patient (<30 years old), which may indicate a rapid bone resorption pattern. As it was a single participant, this was deemed insufficient for further analysis of age‐dependent bone remodeling activity in periodontitis patients. However, it would be interesting for future studies to address this focusing on younger participants.

Transcriptional differences regarding the maturation of periodontal granulation tissue over time in the study reinforce the notion that the beneficial effects of NSPT continue to be expressed beyond standard periods of monitoring before deciding on surgical intervention.^43^ Correspondingly, KEGG analysis revealed pathways involving vascular proliferation and cellular differentiation. The reported pathways pertaining to diabetic complications highlight the shared genetic interactions between diabetes and periodontitis, specifically from an inflammatory perspective.^44^

Detection of DEG based on sex differences reinforces the increasing relevance of the biological variable in periodontitis. Published studies have reported greater severity of periodontitis with poorer treatment response in males compared to females.^45^, ^46^ It has been speculated that males mount a more exaggerated immune response against periodontitis‐initiating pathogens.^47^ Sexual dimorphism in immune response under inflammatory conditions has been reported to influence the chemotactic activity of neutrophils and leukocytes in response to microbial challenge.^48^ Additionally, the distinct genetic makeup of males and females is thought to be capable of modifying pattern recognition mechanisms and intracellular immune signaling pathways.^47^

From the annotated DEG, *CD24* was identified as the most significantly expressed. *CD24*‐derived sialoglycoprotein exhibits immunoregulatory characteristics capable of modulating the effective assembly of tight junction complexes in the epithelium. An increase in inflammatory cytokines has been hypothesized to elevate the expression of *CD24*, which could be the case in severe periodontitis.^49^

The exploratory approach of utilizing next‐generation sequencing has provided valuable information on what is routinely encountered in conventional periodontal surgical procedures but could be strengthened with a higher number of samples for more meaningful data interpretation. A limitation of this study is the lack of controls as it adopted a convenience sampling approach, whereby only samples with suitable RNA quality were analyzed. Although there was no specific intention to harvest samples during NSPT, a longitudinal transcriptional comparison could have been made between granulation tissue prior to NSPT and post NSPT/prior to surgery, as well as with healthy noninflamed gingival tissue. Although a stringent harvesting protocol was implemented, the inclusion of gingival epithelial tissue cannot be completely ruled out even if a sample was observably predominantly granulation tissue. Time‐course comparison can be improved with the expansion of analysis utilizing more timepoints following NSPT. While considerable care and attention was given to excluding lining epithelium from the samples, complete exclusion could not be guaranteed. Although bulk RNA sequencing has its advantages for a comprehensive overview of transcriptome analysis, accurate signals navigating specific and perhaps novel cellular pathways from a unique cell population or cell category can be concealed by an average gene expression profile from the sequencing protocol.^50^

## 5 CONCLUSION

This study has demonstrated that periodontal granulation tissue exhibits a phenotype of chronic wound tissue that has undergone significant remodeling accompanied with immunomodulatory properties. Within the limits of this study, the time‐dependent comparison has revealed the tissue's capacity to differentiate beyond traditional re‐evaluation timeframes post NSPT, but its full differentiation potential remains unknown. It can be speculated that long‐term healing seems to be related to increased collagen maturation and osteogenesis. However, this exploratory finding needs further testing. Additional research will be needed to deepen our understanding of the differentiation capacity of periodontal granulation tissue with respect to time, especially in regard to healing response post NSPT. Males seem to display an expression signature indicative of a more pronounced immune response, which may contribute to greater severity of disease manifestation, but the effects of sexual dimorphism also will require further investigation.

## AUTHOR CONTRIBUTIONS

Mandeep Ghuman and Luigi Nibali contributed to the conception and design of the study. Ye Han Sam, Mandeep Ghuman, Giuseppe Mainas, and Dewi R. Owen were involved in data collection and data analysis. Ana J. Caetano, Mandeep Ghuman, and Ye Han Sam were involved in data interpretation. Mandeep Ghuman and Ye Han Sam drafted the manuscript. Luigi Nibali, Mandeep Ghuman, Ye Han Sam, Ana J. Caetano, Dewi R. Owen, and Giuseppe Mainas critically reviewed the manuscript and gave final approval for the version to be published.

## CONFLICT OF INTEREST STATEMENT

The authors declare no conflicts of interest.

## Data Availability

The data that support the findings of this study are available on request from the corresponding author. The data are not publicly available due to privacy or ethical restrictions.

## Footnotes

* DNA/RNA Shield; Zymo Research, Irvine, California, USA.

† Quick‐RNA Miniprep Kit; Zymo Research, Irvine, California, USA.

‡ NanoDrop2000; Thermo Fisher Scientific, Waltham, Massachusetts, USA.

§ 4200 TapeStation; Agilent Technologies, Santa Clara, California, USA.

‖ Illumina NextSeq 2000; Illumina, San Diego, California, USA.
